# Comparison between integrated backscatter intravascular ultrasound and 64-slice multi-detector row computed tomography for tissue characterization and volumetric assessment of coronary plaques

**DOI:** 10.1186/1476-7120-10-33

**Published:** 2012-08-06

**Authors:** Takahiko Yamaki, Masanori Kawasaki, Ik-Kyung Jang, Owen Christopher Raffel, Yoshiyuki Ishihara, Munenori Okubo, Tomoki Kubota, Arihiro Hattori, Kazuhiko Nishigaki, Genzou Takemura, Hisayoshi Fujiwara, Shinya Minatoguchi

**Affiliations:** 1 Department of Cardiology, Gifu University Graduate School of Medicine, 1-1 Yanagido, Gifu, 501-1194, Japan; 2 Cardiology Division, Massachusetts General Hospital and Harvard Medical School, Boston, MA, USA

**Keywords:** Computed tomography, Integrated backscatter, Intravascular ultrasound, Coronary plaque

## Abstract

**Background:**

The purpose of this study was to determine the cut-off values of Hounsfield units (HU) for the discrimination of plaque components and to evaluate the feasibility of measurement of the volume of plaque components using multi-detector row computed tomography (MDCT).

**Methods:**

Coronary lesions (125 lesions in 125 patients) were visualized by both integrated backscatter intravascular ultrasound (IB-IVUS) and 64-slice MDCT at the same site. The IB values were used as a gold standard to determine the cut off values of HU for the discrimination of plaque components.

**Results:**

Plaques were classified as lipid pool (n =50), fibrosis (n =65) or calcification (n =35) by IB-IVUS. The HU of lipid pool, fibrosis and calcification were 18 ± 18 HU (−19 to 58 HU), 95 ± 24 HU (46 to 154 HU) and 378 ± 99 HU (188 to 605 HU), respectively. Using receiver operating characteristic curve analysis, a threshold of 50 HU was the optimal cutoff values to discriminate lipid pool from fibrosis. Lipid volume measured by MDCT was correlated with that measured by IB-IVUS (r =0.66, p <0.001), whereas fibrous volume was not (r =0.21, p =0.059).

**Conclusion:**

Lipid volume measured by MDCT was moderately correlated with that measured by IB-IVUS. MDCT may be useful for volumetric assessment of the lipid volume of coronary plaques, whereas the assessment of fibrosis volume was unstable.

## Introduction

Enhanced multi-detector row computed tomography (MDCT) is a promising minimally-invasive method for detecting coronary artery disease. This method uses low radiation and requires the intravenous injection of contrast medium. The accuracy of MDCT for evaluating the degree of stenosis in coronary arteries was established in previous studies by direct comparison with angiography [1-6]. However, the ability of MDCT to characterize the tissue components of coronary plaques has been controversial, with some studies showing that MDCT produced results that were similar to conventional intravascular ultrasound (IVUS) [7,8], whereas other studies found that MDCT was not as accurate as IVUS [9,10]. Although MDCT has the potential for discriminating plaque components, the validity of this method in the clinical setting will depend upon development of objective and quantitative methods to analyze MDCT images.

Recently, many techniques for the tissue characterization of plaque composition have been developed using IVUS [11,12]. We previously reported that integrated backscatter (IB)-IVUS had with high sensitivity and specificity (90-95 %) for the characterization of plaque tissue components using histology as a gold standard [13,14]. The reliability and the usefulness of IB-IVUS have been established in many reports [13-17].

The purpose of the present study was [1] to determine the cut-off values of Hounsfield units (HU) for the discrimination of plaque components using IB values as a gold standard and [2] to evaluate the feasibility of measurement of the volume of lipid pool and fibrosis using MDCT.

## Methods

### Study protocols

We enrolled 150 consecutive patients. Inclusion criteria were patients with stable angina pectoris, who were undergoing percutaneous coronary intervention (PCI), angina-unrelated lesions with moderate stenosis in which calcification did not preclude quantitative assessment by IVUS or MDCT and absence of side branches between the proximal and distal portions of the lesion. The plaques analyzed in this study had to be more than 20 mm from the lesion that was targeted for intervention. Patients with unstable angina or myocardial infarction within the previous three months were excluded. The final enrollment included 125 patients (testing study: 45 patients, validation study: 80 patients). Tissue characterization was performed at each site by IB-IVUS (IB-IVUS, YD Co., Ltd., Nara, Japan) within one week of MDCT imaging. The protocol was approved by the institutional ethics committees, and informed consent was obtained from each patient.

### Data acquisition of CT coronary angiography

Patients took isosorbide dinitrate just before MDCT imaging for the prevention of coronary spasm. MDCT imaging was performed with a 64-slice CT scanner (Light Speed VCT, GE Healthcare, Waukesha, Wisconsin). Images were acquired with a gantry rotation time of 350 ms, 64 x 0.625 mm-slice collimation, tube current of 430 mA, and a tube voltage of 120 kV. Contrast agent (Iopamidol, Iodine 370 mg/ml, Iopamiron, Schering) was injected intravenously at a flow rate of 4 ml/s when HU of descending aorta became 50 HU, followed by a 30 ml saline solution chaser bolus. Image reconstruction was retrospectively gated to the ECG. The position of the reconstruction window within the cardiac cycle was individually chosen to minimize motion artifacts. All acquired data were transferred to a computer workstation (Advantage Workstation 4.3, General Electronic), and reconstructed by the half-reconstruction method. The effective slice thickness was 0.625 mm, and the reconstruction increment was approximately 0.5 mm.

### Comparison between MDCT images and IB-IVUS images

Conventional IVUS images and ultrasound signals were acquired using an IVUS imaging system (Clear View, Boston Scientific, MA) with a 40 MHz intravascular catheter. During IVUS imaging, we administered an intra-coronary optimal dose of isosorbide dinitrate before the measurements to prevent of coronary spasm. IB-IVUS images were captured at an interval of 0.5 mm using a motorized pull-back system in each plaque. IB values were calculated as previously described and our definition of IB values for each histological category was determined by comparison with histological images as reported in our previous study [16]. In our previous report, an IB value of ≤ −49 dB was the most reliable cutoff point for discriminating lipid pool (90 % sensitivity, 92 % specificity) and fibrosis (94 % sensitivity, 93 % specificity) and an IB value of >−29 dB was the most reliable cutoff point for discriminating calcification (95 % sensitivity, 99 % specificity) and fibrosis [16].

**Figure 1:**
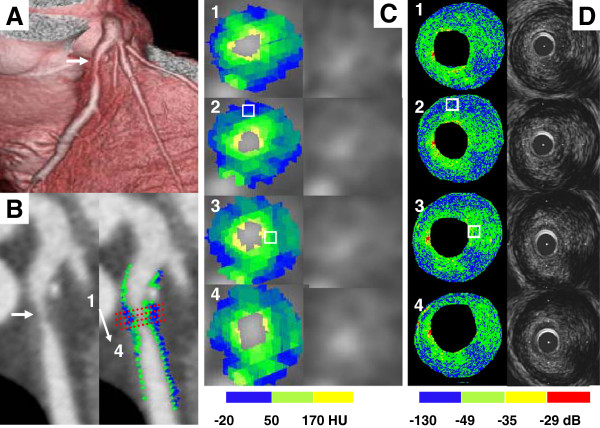
**(A) A volume-rendering image of the left anterior descending artery (arrow).** A plaque with intermediate stenosis. (**B**) (Left) Curved multiplaner reconstruction image shows intermediate stenosis in the proximal portion of left anterior descending artery (arrow). (Right) Color-coded curved multiplaner reconstruction image of coronary plaque. (**C**) (Left) Color-coded cross-sectional multiplaner reconstruction image. (Right) Gray-scale multiplaner reconstruction images. Numbers that identify the images correspond to the cross-section in (**B**). Regions of interests (white square, 0.5 x 0.5 mm) with homogenous tissue components were selected on IB-IVUS images, and were used to set the corresponding region of interests on cross-sectional multiplaner reconstruction images of multidetector computed tomography. (**D**) (Left) Integrated backscatter intravascular ultrasound color-coded maps. (Right) Gray-scale intravascular ultrasound images. Numbers that identify the images correspond to the cross-section in (**B**).

To ensure that the same coronary sections were always compared by IB-IVUS and MDCT, we used the distance from side branches and bifurcation points as reference markers based on the IVUS pullback rate of 0.5 mm/sec. Longitudinal reconstruction of IVUS and MDCT images was used to identify the same corresponding coronary sections (Figure 1). In the testing set (n = 45 lesions), we set a region of interest (ROI) (0.5 x 0.5 mm) in a homogenous tissue component in the reconstructed IB-IVUS images after determining the corresponding cross-sections (Figure 1). The region of interests (ROIs) selected in the reconstructed MDCT images were the same as those selected in the IB-IVUS images, and the HU were compared with the IB values. The one observer independently set ROIs on MDCT images that were same lesions as IB-IVUS images, and another observer independently measured HU of the ROIs.

In the validation set (n = 80 lesions), lipid volume was calculated using the cutoff value that was determined in the testing study for discriminating lipid pool from fibrosis by the following formula: lipid volume (mm^3^) = [plaque (intima + media) area _1_ (mm^2^) x relative lipid area _1_ (%) + plaque area _2_ (mm^2^) x relative lipid area _2_ (%) + ……. + plaque area _n_ (mm^2^) x relative lipid area _n_ (%)] / 100 x 0.5, where n is number of lesion cross-sections analyzed. Fibrous volume was calculated in a similar manner. Calcification volume was also calculated in a similar manner in 49 lesions.

### Reproducibility and reliability of HU measurement

We determined interobserver variability of HU using 20 randomly-selected cross-sections that were measured by two observers in a blinded way. The two observers independently set ROIs on MDCT images that were same cross-sections as IB-IVUS images, and measured HU of the ROIs. Likewise, we determined intraobserver variability of HU using 20 randomly-selected cross-sections that were measured twice by one observer with a 7-days interval between the two measurements.

### Statistical analyses

Continuous values were expressed as the mean ± one standard deviation. Categorical data were summarized as percentages. Receiver operating characteristic (ROC) curves were used to determine the cutoff points for differentiating each tissue component by MDCT. Linear regression analysis was performed to determine the relationship between the volume of lipid pool or fibrosis measured by IB-IVUS and MDCT, and Bland-Altman plots were constructed. Reclassification analysis was conducted by examining the net reclassification improvement (NRI) statistics [18]. A p-value of <0.05 was considered statistically significant. Statistical analyses were performed using Stat View version 5.0 (SAS Institution Inc, Cary, NC, USA).

## Results

### Patient characteristics

A total of 125 patients with stable angina underwent IB-IVUS analysis in non-target lesions without any complications. All patients completed MDCT imaging using contrast medium (67 ± 4 mL) without any associated major events or complications. There were no clinical events within the one week between IB-IVUS and MDCT. The patients’ characteristics in the testing and validation studies are shown in Table 1.

**Table 1:** Patient Characteristics

	**Testing study (n = 45)**	**Validation study (n = 80)**
Sex, n (%)		
Men	36 (80)	68 (87)
Age, y	67 ± 8	69 ± 9
Body mass index, (kg/m^2^)	23.1 ± 3.5	23.4 ± 3.9
Heart rate, (beats/minute)	71 ± 11	68 ± 13
Clinical history, n (%)		
Prior myocardial infarction	6 (13)	18 (14)
Hypertension	32 (71)	63 (80)
Dyslipidemia	16 (36)	35 (44)
Current smoker	5 (11)	16 (20)
Diabetes mellitus type 2	7 (16)	19 (24)
Medications, n (%)		
Antiplatelet medication	45 (100)	80 (100)
Statin	15 (33)	33 (41)
Nirates	25 (56)	53 (66)
Calcium channel blockers	33 (73)	61 (76)
Beta-blockers	16 (36)	30 (38)
Insulin	3 (7)	11 (14)
ACE inhibitors or ARB	34 (76)	53 (66)
Laboratory parameters (mg/dl)		
Total cholesterol	201 ± 29	193 ± 34
Triglycerides	147 ± 67	165 ± 99
HDL cholesterol	49 ± 12	45 ± 11
LDL cholesterol	125 ± 26	116 ± 25
HbA1c	6.2 ± 1.0	6.3 ± 1.1
Lesions, n (%)		
Left anterior descending branch	21 (44)	32 (40)
Left circumflex branch	10 (22)	18 (23)
Right coronary artery	14 (31)	30 (37)

Values are mean ± SD. Numbers in parenthesis are percentage. SA: stable angina. ACS: acute coronary syndrome. ACE: Angiotensin converting enzyme. ARB: Angiotensin II receptor blockers. LDL: Low-density lipoprotein. HDL: High-density lipoprotein.

### Reproducibility and reliability of HU measurement

The interobserver correlation coefficient and mean differences in HU were 0.97 and 1.3 ± 2.6 HU, respectively. The intraobserver correlation coefficient and mean differences in HU were 0.96 and 1.0 ± 2.0 HU, respectively. The inter-observer agreement of plaque components (lipid pool, fibrosis and calcification) by MDCT without cutoff values was 0.66.

### Cut-off values for the discrimination of tissue components

**Figure 2:**
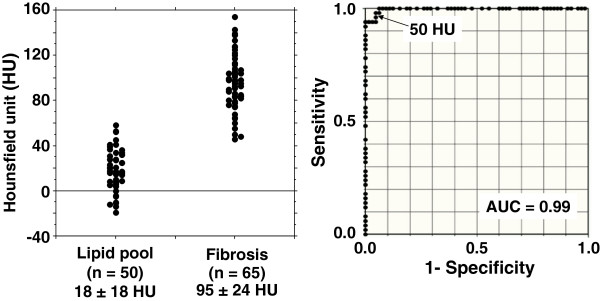
**(A) Comparison between the Hounsfield units of lipid pool and fibrosis.** (**B**) Receiver operating characteristic curves (ROC) analysis based on the Hounsfield units. ROC curves analysis indicated that 50 Hounsfield units measured by multidetector computed tomography was the most reliable cutoff value for detecting lipid pool. AUC: area under the curve.

In the testing set, a total of 150 homogeneous lesions (0.5 x 0.5 mm) in 45 coronary arteries were diagnosed from the IB-IVUS images as lipid pool (n = 50), fibrosis (n = 65) or calcification (n = 35) according to IB values. The HU in the corresponding ROIs (0.5 x 0.5 mm) for lipid pool, fibrosis and calcification, were 18 ± 18 HU (−19 to 58 HU), 95 ± 24 HU (46 to 154 HU) and 378 ± 99 HU (188 to 605 HU), respectively. The distributions of HU for lipid pool and fibrosis are shown in Figure 2. Based on ROC curve, analysis 50 HU was determined as the cutoff value for discriminating lipid pool from fibrosis. There was no overlap between the HU of fibrosis and calcification. Therefore, we provisionally used a cutoff value of 170 HU that was between fibrosis and calcification, since the maximum HU of fibrosis was 154 HU and the minimum HU of calcification was 188 HU. Using these cutoff values, the NRI for the tissue characterization of coronary components was 18.7 % (Table 2).

**Table 2:** Coronary components as diagnosed by models with or without inclusion of Hounsfield unit cutoff values

**Without cutoff values**	**With Hounsfield unit cutoff values**			
	**Lipid pool**	**Fibrosis**	**Calcification**	**Reclassified**
Lipid pool	38	16	0	16
Fibrosis	12	49	0	12
Calcification	0	0	35	0
Total	50	65	35	28

### Comparison between MDCT images and IB-IVUS images

**Figure 3:**
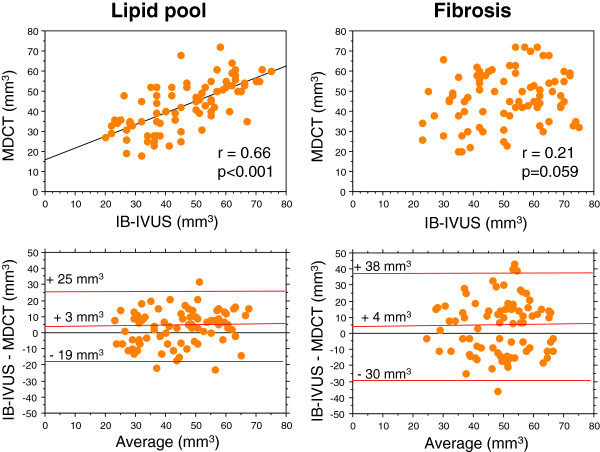
Bland-Altman plots for determining the agreement between integrated backscatter intravascular ultrasound and 64-slice multidetector computed tomography for the measurement of lipid and fibrous volumes in coronary plaques.

**Figure 4:**
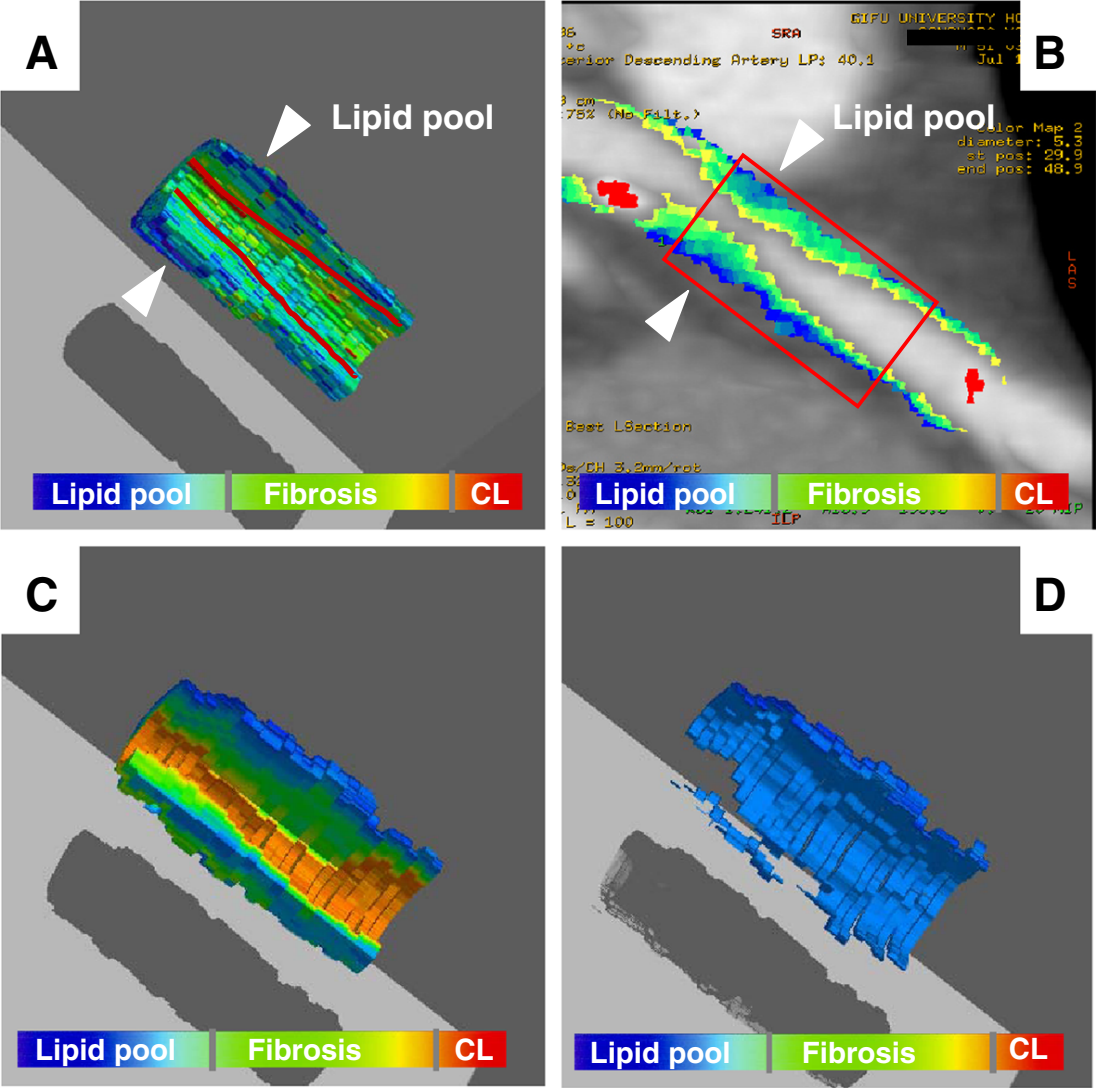
**Representative case of three-dimensional (3D) color-coded maps (68 years old, male).** To reconstruct 3D IB-IVUS color-coded images, the IB-IVUS images were transformed into Cartesian coordinates (64 x 64 pixels) using customized computer software and compared with MDCT images in the Cartesian coordinates (256 x 256 pixels). We then manually excluded the vessel lumen and area outside of the intima in both the 2D IB-IVUS and MDCT images. Three dimensional constructions were automatically performed by computer software (T3D, Fortner Research LLC, Sterling, Virginia). (**A**) 3D color-coded map constructed by integrated backscatter intravascular ultrasound of coronary plaque with intermediate stenosis. (**B**) Color-coded curved multiplaner reconstruction image of coronary plaque. (**C**) 3D color-coded map constructed by multidetector computed tomography for the same lesion as in (**A**). (**D**) 3D color-coded map constructed by multidetector computed tomography that showed only lipid pool in the coronary plaque that was same lesion as (**C**).

At the cutoff value determined by ROC curve analysis in the testing study, lipid volume measured by MDCT was correlated with that measured by IB-IVUS in the validation study (r =0.66, p <0.001). However, fibrous volume measured by MDCT was not correlated with that measured by IB-IVUS (r =0.21, p =0.059) (Figure 3). Calcification volume measured by MDCT was correlated with that measured by IB-IVUS (r =0.63, p <0.001). However, calcification volume measured by MDCT was 2.7 times greater than that measured by IB-IVUS. As shown in Figure 4, the distribution of tissue components based on 3D color-coded maps constructed from MDCT images was similar to the distribution based on 3D maps constructed from IB-IVUS images.

## Discussion

We compared MDCT images with IB-IVUS images and determined cutoff values of HU for discriminating lipid pool from fibrosis, and fibrosis from calcification. We showed that lipid volume measured by MDCT was moderately correlated with that measured by IB-IVUS, whereas fibrous volume was not.

### Comparison between IB values and Hounsfield density

Previous IVUS studies showed that MDCT could accurately characterize the tissue components of coronary plaques by comparison with gray scale IVUS findings [7,19-23]. These studies demonstrated that hypoechoic lesions detected by conventional IVUS had lower HU than those of hyperechoic lesions. Other investigators reported that there was a significant difference in HU between hypoechoic and hyperechoic lesions whereas there was substantial overlap of HU between plaque types [9]. These different results may be because the gray scale IVUS images were evaluated subjectively, and the results may have been influenced by interobserver variability. Another study demonstrated that the ability of MDCT for the discrimination between lipid-rich plaques from fibrous plaques was limited [22]. However, the previous study was based on subjective classification (hypodense, isodense or hyperdense) and relatively low inter-observer agreement of MDCT (κ =0.61). In the present study, we performed an objective quantitative analysis and compared tissue components detected by MDCT with those determined by IB-IVUS. Harada et al. reported that 64-slice MDCT was a promising approach for detection of different types of coronary plaques, but MDCT overestimated low-attenuated plaque and was limited to the determination of low-attenuated plaque volume (r =0.328, p =0.18) [23]. However, in that report, the cutoff (60 HU) between fibrous plaque and low-attenuated plaque was based on the value used in a previous study that was conducted by comparison with gray scale IVUS [20]. In our study, a cutoff of 60 HU resulted in overestimation of low-attenuated plaque, whereas a cutoff of 50 HU, selected by comparison with IB-IVUS, was more accurate to determine the volume of lipid pool (r =0.66, p <0.001). Tanaka et al. reported that mean HU of coronary ruptured plaques was 46.8 HU that was similar to our cutoff value in the present study (50 HU), whereas that of non-ruptured plaques was 73.4 HU [24]. They concluded that 64-slice MDCT might provide a useful tool for the non-invasive detection of plaque rupture. The results of the present study reinforced the previous findings.

### Usefulness of MDCT for tissue characterization of coronary plaques

In the present study, relative fibrous volume measured by MDCT was not correlated with that measured by IB-IVUS, whereas lipid volume measured by MDCT was moderately correlated with that by IB-IVUS. The HU of each tissue component is influenced by its surrounding substances, such as intravascular contrast medium and pericardial fat [10,25]. Lipid pool is generally surrounded by fibrous component, whereas fibrous tissue is surrounded by multiple substances such as pericardial fat or contrast medium [26]. This difference in environment may have contributed to the lack of a correlation of fibrous volume.

Motoyama et al. reported that CT characteristics of plaques associated with ACS included low plaque density (<30 HU) [27]. Kashiwagi et al. reported that mean HU of thin-cap fibroatheroma of coronary plaques was 35.1 HU [28]. This value was similar to that of high risk plaques evaluated by Motoyama et al. In the present study, the cutoff for the discrimination between lipid pool and fibrosis was 50 HU. Taking the three reports into account, a CT value of 50HU was adequate for the differentiation of lipid pool from fibrosis, but not for discrimination between plaques that were associated with ACS and were not associated with ACS. There is the possibility that lower CT values (<30HU rather than 50 HU) indicate the unstable components such as necrotic core. However, IB-IVUS is not able to discriminate between lipid pool and necrotic core. Therefore, it might be difficult to compare the results of the present study with previous studies using histology.

Kitagawa et al. reported that the optimal cutoff values of CT density for predicting hypoechoic lesions evaluated by IVUS was 39 HU, whereas that for lipid pool in the present study was 50 HU [20]. The previous study was performed by comparing hypoechoic and non-hypoechoic lesions that were determined subjectively. In addition, the comparison was performed using relatively large ROIs (one square millimeter), whereas small ROIs (0.5 mm x 0.5 mm) were used in the present study.

Previous studies reported that CT attenuation of coronary atherosclerotic plaques was influenced by intravascular contrast medium [29,30]. CT values of coronary atherosclerotic plaques increased in proportion to the increment of CT values of the coronary lumen up to 250 HU. In contrast, the CT values of coronary atherosclerotic plaques were relatively stable when the values of the coronary lumen were 250–400 HU [29]. The HU of the vessel lumen in the present study was stable (326 ± 55 HU). However, the CT values of the coronary lumen are important when HU is used to characterize tissue components of coronary plaques.

### Clinical implications

We previously reported that the relative volume of lipid pool could be determined using IB-IVUS imaging [13]. However, IB-IVUS is invasive and can only be performed during coronary catheterization, whereas MDCT is minimally invasive and can be applied to the patients who were suspected as angina pectoris. The volume of lipid pool in coronary atherosclerotic lesions can be calculated using 3D color-coded maps constructed from MDCT images. MDCT volumetric analysis of lipid pool may be useful for clinical risk assessment and to provide incremental information on the effectiveness of medications. It was reported that prediction of sudden cardiac death using measurement of coronary calcification by MDCT are distinct methods of assessing risk for sudden cardiac death [31]. Measurement of lipid pool by MDCT may be promising methods of assessing risk for coronary artery disease.

### Study limitations

There are several limitations of the present study. First, calcification is a perfect reflector of ultrasound, causing acoustic shadowing that is typical in IVUS images. The ultrasound signals that are not able to penetrate or pass through the calcified layer are reflected back towards the transducer. Therefore, an accurate calculation of calcified area and volume is not possible using ultrasound. Likewise, a beam-hardening action by calcification that is called “partial volume effect”, hinders rigorous evaluation of the calcified volume by MDCT. Second, we excluded the area of the artifact due to the guidewire from the IB-IVUS analyses. Therefore, the guidewire artifact and calcification interfere with a rigorous calculation of the area and volume of each component. Comparison between IB values and HU of unstable plaques including thin cap fibroatheroma in patients with acute coronary syndrome is required. Third, square shaped ROI (0.5 x 0.5 mm) might be too large to enclose homogenous plaque components in human coronary plaques. Improvement in resolution of MDCT would be expected in the future. Finally, the IB-IVUS remains a research tool which does not have clinical utility. The use of IB values that was surrogate as the gold standard did not necessarily translate into an accurate analysis of plaque components.

## Conclusions

Using the IB values as a gold standard, lipid volume measured by MDCT was moderately correlated with that measured by IB-IVUS. MDCT may be useful for volumetric assessment of the lipid volume of coronary plaques, whereas the assessment of fibrosis volume was unstable.

## Abbreviations

ACS: Acute coronary syndrome; MDCT: Multi-detector raw computed tomography; IVUS: Intravascular ultrasound; IB: Integrated backscatter; 2D: Two-dimensional; HU: Hounsfield units; PCI: Percutaneous coronary intervention; ROI: Region of interest; ROC: Receiver operating characteristic; 3D: Three-dimensional.

## Competing interests

The authors declare that they have no competing interests.

## Authors’ contributions

TY, OCR and IKJ carried out subject recruitment and analyzed data. MK analyzed data and wrote the manuscript. YI, MO, and TK performed integrated backscatter ultrasound analysis. HF revised manuscript. AH, KN, GT and SM analyzed data. All authors read and approved the final manuscript.

We have no financial or other relations that could lead to conflict of interest.
